# Suprachoroidal hemorrhage followed by swept-source optical coherence tomography: a case report

**DOI:** 10.1186/s12886-018-0881-4

**Published:** 2018-08-20

**Authors:** Kengo Uramoto, Noriaki Shimada, Hiroyuki Takahashi, Hideki Murai, Kosei Shinohara, Kyoko Ohno-Matsui

**Affiliations:** Department of Ophthalmology and Visual Science, Tokyo Medical and Dental University, 1-5-45 Yushima, Bunkyo-ku, Tokyo, 113-8519 Japan

**Keywords:** Suprachoroidal hemorrhage, High myopia, SS-OCT

## Abstract

**Background:**

To report a case of Suprachoroidal Hemorrhage followed by Swept-Source Optical Coherence Tomography.

**Case presentation:**

A 66-year-old woman with a rhegmatogenous retinal detachment in her left eye underwent pars plana vitrectomy. During the intraocular photocoagulation for a retinal tear after fluid-air exchange, a vitreous hemorrhage and suprachoroidal hemorrhage (SCH) developed. The surgical incisions were closed after filling the vitreous cavity with silicone oil. Two weeks later, the hemolyzed hemorrhage was removed, and new silicone oil was injected. After the surgery, a low reflective region was detected near the macula in the swept-source optical coherence tomographic (SS-OCT) images. The low reflective region was caused by the residual hemorrhage. The size of the reflective region gradually decreased and was not present at 3 months. We conclude that SS-OCT can be used to follow the resolution of a suprachoroidal hemorrhage.

**Conclusion:**

SS-OCT can be used to detect and follow the natural course of a suprachoroidal hemorrhage including the absorptive processes.

## Background

A suprachoroidal hemorrhage is a severe complication that can develop during intraocular surgery [1]. It is generally believed that a suprachoroidal hemorrhage develops after a disruption of the posterior ciliary arteries. Ocular hypertension, high myopia, glaucoma, age, high intraocular pressure, arterial sclerosis, diabetes, and aphakia have been reported as preoperative risk factors for developing a suprachoroidal hemorrhage [1]. The intraoperative risk factors are increasing venous return, high intraocular pressure, and vitreous prolapse [2].

The choroidal vessels can be weakened by these risk factors, and when high pressure or stress is applied to increase the transmural venous pressure, the choroidal vessels can be disrupted. These changes are considered to be the cause of a suprachoroidal hemorrhage. In severe cases, the size of the hemorrhage is rapidly increased accompanied by a prolapse of the vitreous and retina. If this happens, the prognosis for the visual acuity is poor. The incidence of a suprachoroidal hemorrhage is 1.9% after vitrectomy [3] and 0.2% after cataract surgery [1, 4].

A search of Medline did not extract any publications showing the course of a suprachoroidal hemorrhage documented by optical coherence tomography (OCT). We report our swept-source OCT (SS-OCT) findings obtained just after the development of a suprachoroidal hemorrhage to its resolution.

## Case presentation

**Fig. 1:**
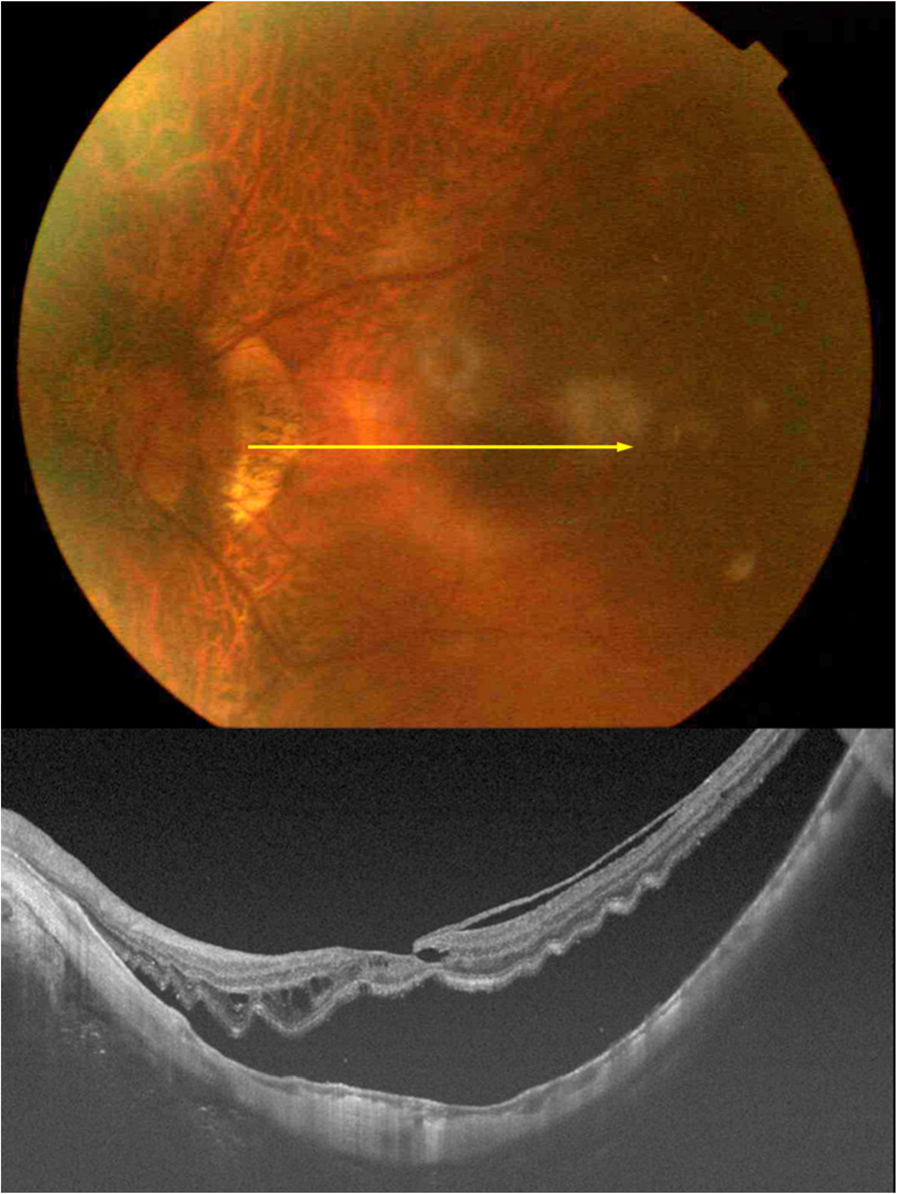
Fundus photograph and swept-source optical coherence tomographic (SS-OCT) image at the initial visit

A 66-year-old woman with reduced vision in her left eye was examined in the Tokyo Medical and Dental University Hospital on June 27, 2015. Her best-corrected visual acuity (BCVA) in her left eye was 20/50, and both eyes were pseudophakic. The refractive error of the left eye was − 1.75 diopters, and the axial length was 25.89 mm. A rhegmatogenous retinal detachment (RRD) was detected by ophthalmoscopy and SS-OCT which extended over the inferotemporal quadrant including macula in her left eye (Fig. 1).

She underwent pars plana vitrectomy on the same day, and during the intraocular photocoagulation for a retinal tear, a sudden choroidal elevation and vitreous hemorrhage occurred. A suprachoroidal hemorrhage was also observed at the site of the trocar insertion. Although we intended to inject silicone oil into the vitreous cavity, a sufficient amount could not be injected because of an obstruction at the trocar insertion site. The surgery was terminated with a closure of the surgical incision.

**Fig. 2:**
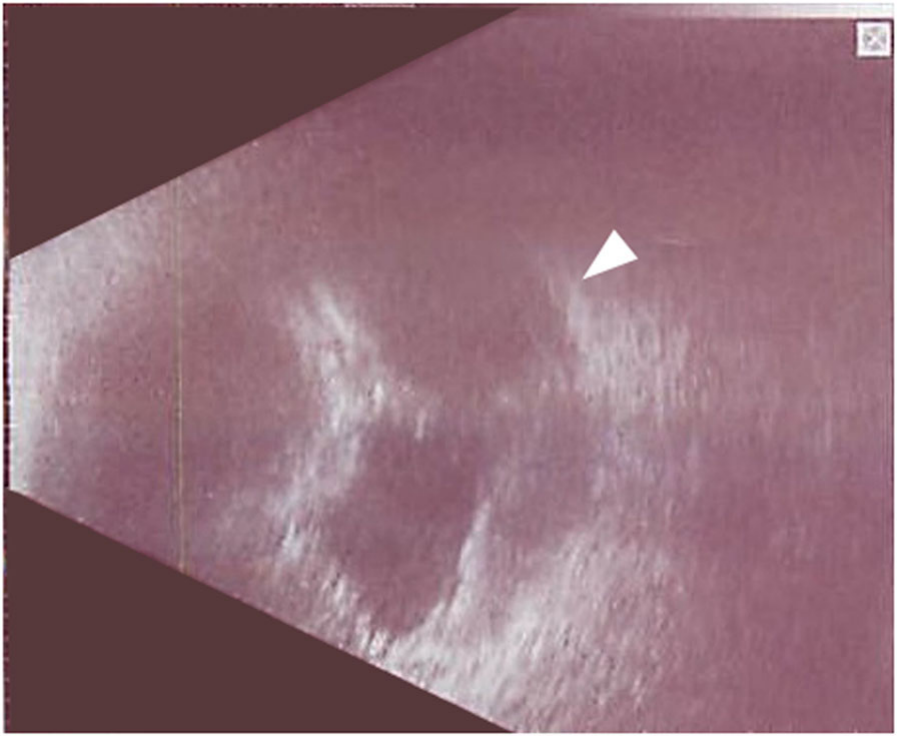
B-scan ultrasound image recorded on postoperative day 1. A dark area was observed in the image which was identified a suprachoroidal hemorrhage (arrow head)

On postoperative day 1, an elevated lesion was detected in the B-scan ultrasound images that was considered to be the suprachoroidal hemorrhage (Fig. 2).

The hemolyzed hemorrhage was removed by scleral fenestration on postoperative day 13. During the surgery, a reduction of choroidal elevation was observed. New silicone oil was injected after removing the original silicone oil.

**Fig. 3:**
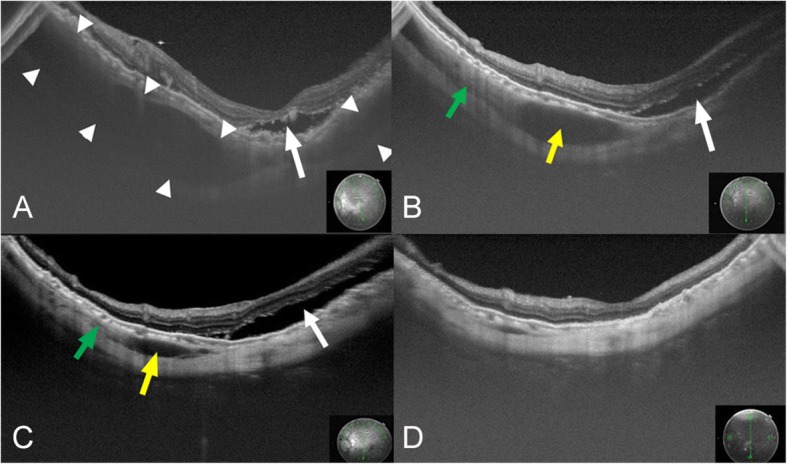
SS-OCT images showing the resolution of a suprachoroidal hemorrhage. On postoperative day 26, a low reflective region can be seen in the suprachoroidal space (between arrowheads) in the SS-OCT image **a** day 26. This was considered to be a suprachoroidal hemorrhage. An elevation of the retina and choroid can be seen at the same region. The low reflective region (yellow arrows) and moderate reflective region (green arrows) are gradually absorbed and not present on postoperative day 73 **b** day 38, **c** day 56, **d** day 73. During the follow-up period, the RRD (white arrows) was still present, and an encircling procedure with silicone oil tamponade was performed on postoperative day 63. The hemorrhage was slowly absorbed and was not detected on postoperative day 73

Postoperatively, the resolution of the suprachoroidal hemorrhage was followed in the SS-OCT images (Fig. 3). However, the RRD on the inferior quadrant remained, and a scleral encircling procedure was performed on day 63, and silicone oil was reinjected to tamponade the retina. On day 73 after the RRD surgery, the retina was reattached, and the suprachoroidal hemorrhage was not present in the SS-OCT images.

At 3 months after the most recent surgery, the patient’s BCVA is 20/200, and the silicone oil was still present. A future surgery is planned to remove the silicone oil.

## Discussion

This case report shows the process of a gradual absorption of a fresh hemorrhage in the suprachoroidal space by SS-OCT. A suprachoroidal hemorrhage is observed as low to moderate reflective regions in SS-OCT images. Generally, a subretinal hemorrhage is observed as high reflective region especially next to the retina in the OCT images [5]. We suggest that the low reflective region in our case was due to the hemolyzed liquid hemorrhage component and the moderate reflective region was the non-liquid component of the hemorrhage. It might be possible that the phase when the low reflective region is observed might be the appropriate timing for removing the suprachoroidal hemorrhage.

There are some diseases which shows the similar OCT findings to suprachoroidal hemorrhage, such as choroidal hemangioma, choroidal malignant melanoma, and choroidal nevus. The choroidal hemangioma exhibits a mottled high reflection image on the tumor surface due to the reflection of the blood vessel wall. The choroidal malignant melanoma/choroidal nevus exhibit an internal homogeneous low-reflection image similarly to suprachoroidal hemorrhage, but it is easy to distinguish the suprachoroidal hemorrhage from these diseases because of the specific course of the onset. In addition, the suprachoroidal location of the lesions is useful for making differential diagnosis from choroidal lesions.

Because of the deep penetration into tissues, SS-OCT is considered a powerful tool for the management of postoperative suprachoroidal hemorrhage. In spite of the limitation of a single case report, we believe it would be beneficial for clinicians to know the change of OCT findings in the course of the resolution of a suprachoroidal hemorrhage. Further studies including a larger number of patients are considered necessary to confirm the value of SS-OCT for a tool to visualize the course of this serious complication. In spite of the limitation of single case report, we believe it would be beneficial for clinicians to know the change of OCT findings in the course of the resolution of a suprachoroidal hemorrhage.

## Conclusion

We conclude that SS-OCT can be used to detect and follow the natural course of a suprachoroidal hemorrhage including the absorptive processes.

## Abbreviations

BCVA: Bbest-corrected visual acuity
OCT: Optical coherence tomography
RRD: Rhegmatogenous retinal detachment
SS-OCT: Swept-source OCT
